# Ginseng Polysaccharides Protect Against Endoplasmic Reticulum Stress-Induced Damage via PI3K/Akt Signalling Pathway in Bovine Ovarian Granulosa Cells

**DOI:** 10.3390/cells15020172

**Published:** 2026-01-17

**Authors:** Hongjie Wang, Yi Fang, Lei Huang, Xu Yang, Xin Ma, Yang Lyu, Guo Jing, He Ding, Hongyu Liu, Wenfa Lyu

**Affiliations:** 1 Key Laboratory of Animal Production, Product Quality and Security, Ministry of Education, Jilin Agricultural University, Changchun 130118, China; 13287712312@163.com (H.W.); fangyi@iga.ac.cn (Y.F.); 13231831867@163.com (L.H.); worronyang25@yeah.net (X.Y.); maxin3202@163.com (X.M.); 15134378707@163.com (Y.L.); jgchn@163.com (G.J.); dinghe1103@126.com (H.D.); 2 Key Laboratory of Utilization and Protection of Beef Cattle Germplasm Resources, Jilin Agricultural University, Changchun 130118, China; 3 Jilin Provincial International Joint Research Center of Animal Breeding & Reproduction Technology, Jilin Agricultural University, Changchun 130118, China

**Keywords:** ERS, ginseng polysaccharides, PI3K/Akt pathway, ovarian granulosa cells, necroptosis

## Abstract

**Highlights:**

**What are the main findings?**

- Ginseng polysaccharides protect ovarian granulosa cells by alleviating endoplasmic reticulum stress and inhibiting subsequent necroptosis via the RIPK3/MLKL pathway.
- The polysaccharides restore cellular proliferation and estrogen synthesis compromised by ERS, primarily through activation of the PI3K/Akt signalling pathway, a mechanism corroborated by in vivo studies.

**What are the implications of the main findings?**

- The study identifies ginseng polysaccharides as a promising natural agent for protecting ovarian function, offering a potential preventive or therapeutic strategy against ovarian disorders driven by endoplasmic reticulum stress and necroptosis, such as premature ovarian insufficiency.
- It establishes a clear molecular pathway where these polysaccharides alleviate ER stress, inhibit the downstream RIPK3/MLKL-mediated necroptosis, and activate pro-survival PI3K/Akt signalling to restore cell health, providing a validated target for future interventions.

**Abstract:**

Necroptosis and dysfunction of ovarian granulosa cells are major contributors to follicular atresia and reduced fertility in cattle, processes that are closely associated with endoplasmic reticulum stress (ERS). Ginseng polysaccharides (GPSs) are known to reduce ER stress, display anti-inflammatory properties, and modulate reproductive function; however, whether GPS can protect against granulosa cell injury and the underlying mechanisms remain unclear. To address this gap, this study aimed to investigate the protective effects of GPS on ERS-induced bovine granulosa cell damage and to elucidate the associated mechanisms. An ERS model was established in bovine granulosa cells using tunicamycin (Tm), and cellular responses were evaluated via flow cytometry, ELISA, and EdU assays. Further, a mouse model was used to validate the protective effects of GPS against Tm-induced ovarian injury. The results showed that 40 μg/mL of GPS significantly alleviated ERS-induced granulosa cell damage, inhibited necroptosis, and mitigated ERS. Moreover, using the PI3K/Akt pathway inhibitor LY294002, we demonstrated that the inhibitor antagonized the effects of GPS, indicating that GPS promotes granulosa cell proliferation and restores estrogen secretion via activating the PI3K/Akt pathway. In vivo experiments further confirmed that GPS effectively attenuates ERS-induced ovarian damage in mice. Collectively, these findings reveal that GPS improves granulosa cell function and ovarian tissue integrity by modulating the ERS network and the PI3K/Akt pathway, yielding a theoretical basis for preventing follicular atresia and enhancing reproductive efficiency in cattle.

## 1. Introduction

Follicular growth and development play an irreplaceable role in maintaining reproductive performance in cattle. In the bovine ovary, granulosa cells (GCs), as the primary structural component of the follicle, are central to maintaining normal follicular structure and function. They create a microenvironment conducive to oocyte development, maturation, and ovulation by providing essential regulatory factors such as hormones and growth factors [1,2,3]. Previous research indicates that the expansion of ovarian granulosa cells helps inhibit follicular atresia and supports the production of sex steroids during follicular development [4]. Furthermore, ovarian GCs release bioactive molecules such as estradiol (E2) through autocrine and paracrine pathways, thereby participating in the fine regulation of oocyte maturation [5,6]. However, during follicular development, approximately 99% of follicles undergo atresia due to maturation failure. Wu et al. [7] confirmed that necroptosis is a central cellular event in follicular atresia. Necroptosis represents a regulated mode of cellular demise driven by the signalling pathway involving receptor-interacting protein kinase 3 (*RIPK3*) and mixed-lineage kinase domain-like (*MLKL*) and is characterized by the liberation of inflammatory mediators [8,9].

During follicular growth and development, persistent imbalance of endoplasmic reticulum (ER) homeostasis can lead to the accumulation of unfolded and misfolded proteins, thereby inducing ER stress (ERS) and activating the unfolded protein response (UPR) [10,11]. ERS is fundamentally involved in the modulation of reproductive functions in female mammals, including the modulation of the follicular microenvironment (e.g., follicular atresia and luteal regression), thereby providing a molecular basis for the cyclical changes in ovarian function [12,13]. As a key adaptive mechanism in response to ERS, UPR activation depends on three core transmembrane sensors, *IRE1*, *PERK*, and *ATF6*, which regulate gene expression related to secretory pathways and determine cell fate [14,15]. When the severity of ERS surpasses the adaptive threshold of the cells, the UPR transitions from a protective mechanism to one that facilitates apoptosis, ultimately driving follicular atresia through the induction of programmed death in granulosa cells and other follicular cell populations [16]. Previous studies have shown that ERS can mediate necroptosis of bovine ovarian GCs via the downstream PERK–eIF2α–ATF4 pathway [17]. Additionally, chronic ERS can lead to GC dysfunction, inhibit GC proliferation, and suppress the expression of key steroidogenic molecules such as cytochrome P450 aromatase (*CYP19A1*) and steroidogenic acute regulatory protein (*StAR*) [18,19]. Therefore, identifying compounds that effectively alleviate ERS in ovarian GC damage models holds significant potential for treating reproductive disorders associated with abnormal follicular atresia.

Ginseng, a traditional Chinese medicinal herb, has garnered substantial scientific interest because of its wide range of therapeutic properties. Ginseng polysaccharides (GPSs), core bioactive components extracted from ginseng, are high-molecular-weight polysaccharides with extensive pharmacological properties, including metabolic regulation, antioxidant, anti-inflammatory, and immunomodulatory effects [20,21]. In non-reproductive tissues, GPS has been reported to alleviate ERS-related damage by activating the phosphatidylinositol 3-kinase (*PI3K*)/protein kinase B (*Akt*) signalling pathway and modulating the PERK–eIF2α–ATF4 axis [22]. In the field of reproduction, GPS has been shown to reduce the weak piglet rate and stillbirth rate in sows [23] and to activate the PI3K/Akt signalling pathway in mouse testicular tissues, thereby promoting steroid hormone synthesis in Leydig cells [24]. Nevertheless, whether GPS enhances reproductive performance in female ruminants has not yet been fully elucidated, especially regarding its potential to alleviate ovarian granulosa cell injury through modulation of the PI3K/Akt axis and signalling pathways associated with ERS.

This study aims to investigate the potential of ginseng polysaccharides in mitigating ERS-induced ovarian damage. We combine mechanistic studies of bovine granulosa cells with phenotypic validation using a mouse model to comprehensively assess the efficacy and mode of action of GPS. The results provide foundational evidence supporting the future development of GPS-based interventions for ERS-related ovarian disorders.

## 2. Materials and Methods

### 2.1. Animals and Treatments

In this experiment, a total of 24 specific-pathogen-free (SPF) Kunming mice (6–8 weeks old, body weight ranging from 34 to 40 g) were acquired from Liaoning Longevity Biological Technology Co., Ltd., Shenyang, Liaoning, China. The mice were randomly assigned to 3 experimental groups using a randomized block design based on body weight and age, with 8 mice per group (*n* = 8) to ensure balanced baseline characteristics among groups. All procedures involving animals, including their maintenance and experimental handling, were performed in compliance with the regulatory standards set by Jilin Agricultural University (Approval ID: 20230824001). After one week of acclimatization, the mice were randomly divided into three groups: the Normal Control group (Con, Group 1), the ERS model group (Tm, Group 2), and the Tm + GPS group. GPS was administered at a dosage of 200 mg/kg [22,24], and the ERS inducer tunicamycin (Tm) was administered at a dose of 6 μg/g [25]. The Tm + GPS group received GPS dissolved in 0.9% saline via oral gavage for 7 consecutive days, while the other two groups received 0.9% saline. On day 7, the Tm and Tm + GPS groups were intraperitoneally injected with Tm dissolved in saline, and the Con group was injected with 0.9% saline. Twenty-four hours later, blood was collected from the mice via the retro-orbital venous plexus. After anesthesia was administered, the mice were euthanized through cervical dislocation in accordance with humane procedures, after which their ovarian tissues were collected for subsequent experimental assessments.

### 2.2. Chemicals and Reagents

Ginseng polysaccharides (Cat#: C12N9Y74733) were obtained from Shanghai Yuanye Biotechnology Co., Ltd., Shanghai, China. The PI3K antagonist LY294002 (Cat#: RPM0021) was purchased from ABclonal, Wuhan, China. DMEM/F12 medium (batch number 6124193) was sourced from Gibco, Grand Island, NY, USA. Phosphate-buffered saline (PBS, batch number P16024D04) was obtained from NobleRyder, Beijing, China. SYBR^®^ Premix Ex Taq™ II (Cat#: 820A) was from Takara, Kusatsu, Shiga Prefecture, Japan. Insulin–Transferrin–Selenium (ITS) supplement (Cat#: BL1886A) was purchased from Biosharp, Hefei, China. Penicillin–streptomycin (Cat#: K514KA4196), DEPC (Cat#: K301KA3144), and tunicamycin (Tm, Cat#: A428446-0001) were obtained from Sangong Biotech, Shenyang, China. Prime Script RT Reagent Kit with gDNA Eraser (Cat#: RR047A) was supplied by Takara, Japan. The mouse estrogen (E2) ELISA kit (Cat#: ml063198) was purchased from Mlbio, Dalian, China, and the bovine estrogen (E2) kit (Cat#: JL11602-48T) was from JONLNBIO, Shanghai, China.

The antibodies used in this study included the following: anti-Proliferating Cell Nuclear Antigen (PCNA) (Abcam, Cambridge, UK, Cat#: ab18197), anti-Cyclin-Dependent Kinase 2 (CDK2) (Proteintech, Wuhan, China, Cat#: 6031-1-1g), anti-Phosphoinositide 3-Kinase (PI3K) (Cell Signaling Technology, Danvers, MA, USA, Cat#: 4249), anti-phosphorylated PI3K p85 (p-PI3K) (Cell Signaling Technology, Cat#: 4292), anti-Protein Kinase B (Akt) (Cell Signaling Technology, Cat#: 92725), anti-phosphorylated AKT (p-AKT) (Affinity, Cincinnati, OH, USA, Cat#: AF0832), anti-Glucose-Regulated Protein 78 (GRP78) (Proteintech, Cat#: 11587-1-AP), anti-Inositol-Requiring Enzyme 1 (IRE1) (Proteintech, Cat#: 27528-1-AP), anti-Activating Transcription Factor 6 (ATF6) (Wanleibio, Shenyang, China, Cat#: WL02407), anti-PERK (Protein Kinase R-like ER Kinase) (Proteintech, Cat#: 20582-1-AP), anti-StAR (Steroidogenic Acute Regulatory Protein) (Genescript, Piscataway, NJ, USA, Cat#: 207-220), anti-Cytochrome P450 Family 11 Subfamily A Member 1 (CYP11A1) (Genescript, Cat#: 55-88), anti-Cytochrome P450 Family 19 Subfamily A Member 1 (CYP19A1) (ProSCI, Cat#: 70-306), anti-Receptor-Interacting Protein Kinase 3 (RIPK3) (Santa Cruz, Dallas, TX, USA, Cat#: Sc-374639), anti-Mixed-Lineage Kinase Domain-Like Protein (MLKL) (Proteintech, Cat#: 66675-1-1g), and anti-β-Actin (Proteintech, Cat#: 60008-1-1g).

### 2.3. Ovarian Granulosa Cell (GC) Culture and Treatment

Healthy bovine ovaries were obtained from slaughterhouses in Changchun, China. The ovaries were collected and immediately placed in 37 °C saline containing penicillin (100 IU/mL) and streptomycin (100 mg/mL) and then transported to the laboratory within 4 h. Upon arrival, they were washed five times with saline containing penicillin and streptomycin, after which granulosa cells (GCs) were extracted in a cell culture room. Follicular fluid was aspirated from follicles (3–8 mm in diameter) using a 5 mL syringe and collected into 5 mL conic tube. After centrifugation at 1000 rpm for 5 min, the pellet was washed 2–3 times with PBS. The isolated cells were resuspended and cultured in DMEM/F-12 medium supplemented with 1% Insulin–Transferrin–Selenium (ITS) at 37 °C with 5% CO_2_ [26].

### 2.4. RNA Extraction and Real Time-Quantitative PCR (RT-qPCR)

Total RNA was extracted from collected cells using a TransZol Up High Performance RNA Extraction Kit (TransGen Biotech, Beijing, China) according to the manufacturer’s instructions and then reverse-transcribed to cDNA using PrimeScript™ RT kit (Takara, Tokyo, Japan). All primers employed in this study were generated with Primer Premier 6 and produced by a commercial synthesis service. The primer information is presented in Table 1. Quantitative PCR was carried out, and relative mRNA expression levels were calculated using the 2^−ΔΔCT^ method [17].

### 2.5. CCK-8 Assay

Ovarian granulosa cells were seeded into 96-well plates. After attachment, cells were treated with GPS (dissolved in DMEM with ITS). After 24 h of treatment, CCK-8 (APE×BIO, Houston, TX, USA) solution (CCK-8 reagent: DMEM = 1:50) was used in the dark, followed by incubation for 3 h [27]. Cell viability was assessed by recording the 450 nm absorbance with a microplate reader.

### 2.6. 5-Ethynyl-2′-deoxyuridine (EdU) Assay

Bovine ovarian granulosa cells were incubated in a 6-well plate. After washing with PBS, the medium was replaced with fresh medium containing 10 μmol/L EdU, and the cells were incubated for an additional 2 h. After discarding the medium, the cells were fixed in 4% paraformaldehyde for 15 min at room temperature, rinsed three times with PBS containing 3% BSA, and then permeabilized in 0.3% Triton X-100 for 10–15 min. Following additional washes with PBS, 0.5 mL of Click reaction solution was added to each well, and the cells were incubated for 30 min in the dark. After washing, the cell nuclei were stained with Hoechst 33342 [28]. The cells were observed and imaged under a fluorescence microscope.

### 2.7. Western Blotting Analysis

The protein samples were mixed with loading buffer and denatured by boiling for 10 min. Protein separation was performed using SDS-PAGE. Subsequently, the proteins were transferred onto a nitrocellulose membrane under constant-current conditions using a semi-dry transfer system. The membrane was first treated with a blocking solution for a two-hour period and subsequently exposed to the primary antibody during an overnight incubation. After washing with TBST for a total of 30 min, the membrane was subsequently exposed to the secondary antibody for a one-hour incubation period and washed again with TBST [27]. Protein bands were detected using an ECL chemiluminescence kit and quantified using ImageJ 1.54g software.

### 2.8. Flow Cytometry Assay

For cell cycle analysis, ovarian GCs were collected and stained with propidium iodide (PI). Analysis of cell cycle profiles was carried out with the aid of a flow-cytometric system (ACEA Biosciences, Hangzhou, China). For necroptosis and apoptosis detection, ovarian GCs were resuspended in 1× binding buffer containing FITC-conjugated Annexin V and propidium iodide (PI) solution (BD Biosciences, San Jose, CA, USA) [17]. After incubation in the dark at 37 °C for 15 min, the cells were analyzed by flow cytometry.

### 2.9. Enzyme-Linked Immunosorbent Assay (ELISA) for Estrogen (E2)

For bovine granulosa cell culture supernatants, E2 levels were determined using the bovine estrogen (E2) ELISA kit (Cat#: JL11602-48T, JONLNBIO, Shanghai China), specifically validated for cell culture supernatant matrices. For mouse serum samples (collected from whole blood), E2 levels were measured using the mouse estrogen (E2) ELISA kit (Cat#: ml063198, Mlbio, Shanghai, China), which is validated for serum matrices. All assays were performed strictly following the instructions of manufacturers. After the reaction, the absorbance value of each well was measured at a wavelength of 450 nm using a microplate spectrophotometer. E2 concentrations in samples were calculated based on the standard curve generated by the kit.

### 2.10. Statistical Analysis

Data are expressed as the mean ± standard deviation. Group differences were assessed using one-way ANOVA, with significance defined at * *p* < 0.05, ** *p* < 0.01, and *** *p* < 0.001. Statistical evaluations were conducted in GraphPad Prism 8.0.

## 3. Results

### 3.1. Ginseng Polysaccharides Inhibit Tm-Induced Necroptosis in Ovarian GCs

**Figure 1:**
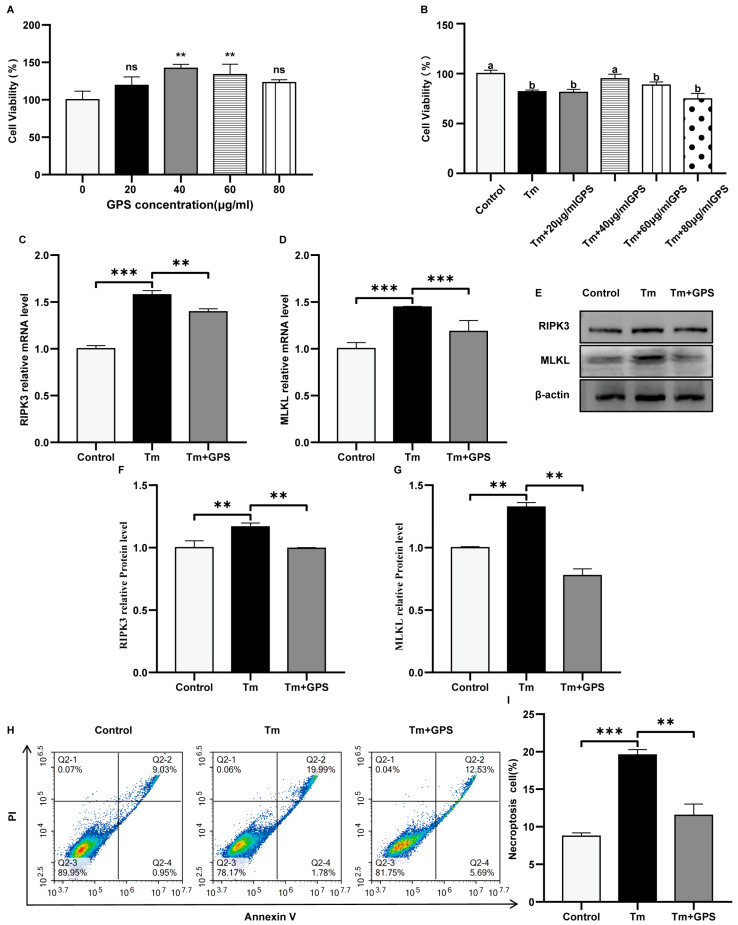
Ginseng polysaccharides inhibit Tm-induced necroptosis in ovarian GCs. (**A**) Viability of ovarian GCs after 24 h treatment with GPS. (**B**) Influence of varying GPS pretreatment doses on the survival of granulosa cells subjected to Tm exposure (5 μg/mL). (**C**,**D**) mRNA levels of necroptosis-related markers. (**E**–**G**) Expression levels of necroptosis-associated proteins. (**H**,**I**) Flow cytometric analysis of the proportion of necroptotic cells (upper-right quadrant). Data are mean ± SD. *n* = 5 independent experiments. One-way ANOVA followed by Tukey’s test. Different lowercase letters represent significant differences between groups (*p* < 0.05). ns *p* > 0.05; ** *p* < 0.01; *** *p* < 0.001.

We first evaluated the viability of bovine ovarian granulosa cells treated with various concentrations of GPS (20, 40, 60, or 80 μg/mL). As shown in Figure 1A, these concentrations were generally non-cytotoxic. The cells were then pretreated with GPS for 24 h, followed by exposure to 5 μg/mL of tunicamycin (Tm) for 10 h. Pretreatment with 40 μg/mL of GPS significantly enhanced the viability of Tm-exposed granulosa cells and mitigated Tm-induced cytotoxicity (Figure 1B). Therefore, 40 μg/mL of GPS was selected for subsequent experiments. RT-qPCR results indicated that exposure to Tm markedly elevated the transcript levels of the ER stress-related necroptosis indicators *RIPK3* and *MLKL* (*p* < 0.05; Figure 1C,D). GPS pretreatment attenuated these increases. Western blot analysis confirmed that Tm markedly increased RIPK3 and MLKL protein levels, which was reversed by GPS pretreatment (*p* < 0.05; Figure 1E–G). Flow cytometric assessment demonstrated that Tm exposure elevated the proportion of cells undergoing necroptosis, whereas pretreatment with GPS markedly diminished this increase (*p* < 0.05; Figure 1H,I). Collectively, these results indicate that GPS inhibits Tm-induced necroptosis in ovarian granulosa cells.

### 3.2. Ginseng Polysaccharides Alleviate Tm-Induced ERS

**Figure 2:**
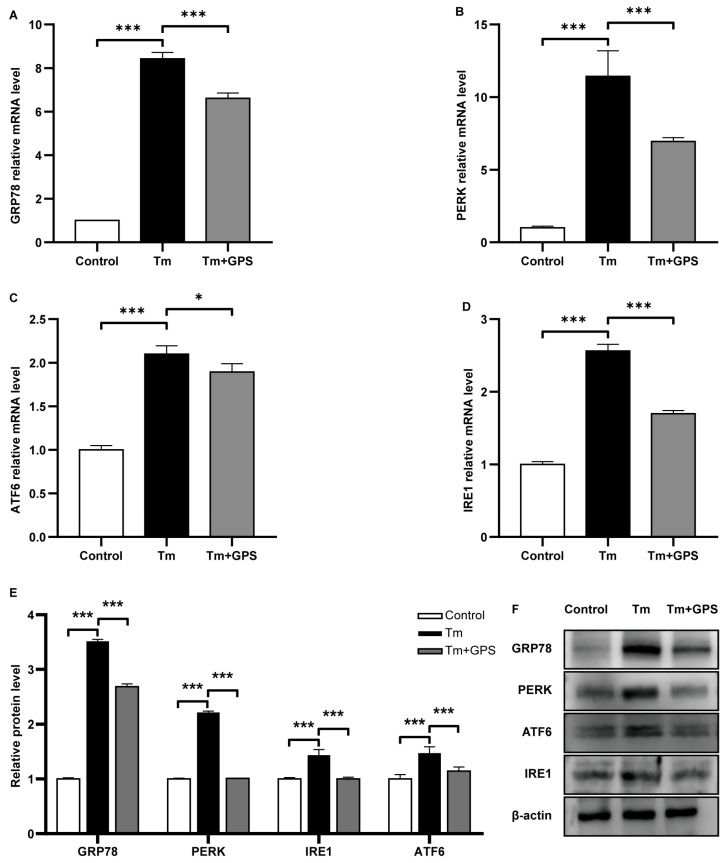
Ginseng polysaccharides alleviate Tm-induced ERS. (**A**–**D**) Relative mRNA levels of ERS-related markers. (**E**,**F**) Expression levels of ERS-associated proteins. Data are mean ± SD. *n* = 5 independent experiments. One-way ANOVA followed by Tukey’s test. * *p* < 0.05; *** *p* < 0.001.

Given that Tm induces necroptosis via ERS-related mechanisms, we next examined ERS-associated markers. RT-qPCR analysis showed that Tm significantly upregulated the mRNA expression of *GRP78*, *PERK*, *ATF6*, and *IRE1* (*p* < 0.05), and GPS treatment suppressed these elevations (Figure 2A–D). Western blot analysis further demonstrated increased protein levels of these ERS markers in the Tm group, which were reversed by GPS pretreatment (*p* < 0.05; Figure 2E,F). These findings indicate that GPS alleviates ERS and inhibits the activation of all three UPR branches.

### 3.3. Ginseng Polysaccharides Alleviate Tm-Induced Impairment of Ovarian GC Proliferation via a PI3K/AKT-Dependent Mechanism

**Figure 3:**
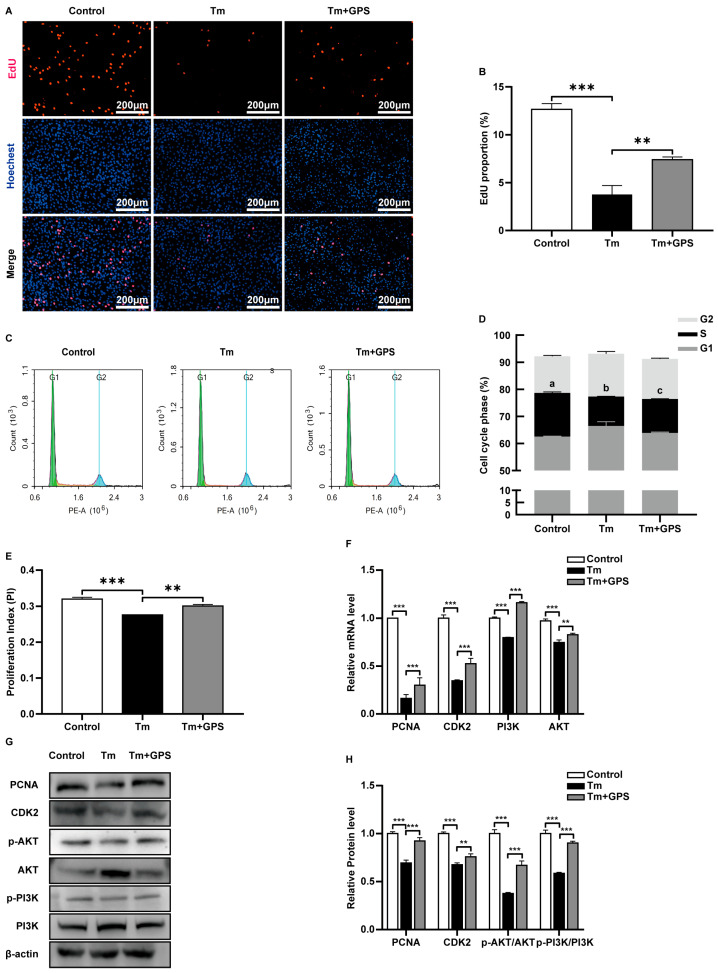
Ginseng polysaccharides alleviate Tm-induced proliferation impairment in ovarian GCs. (**A**) EdU staining of cells followed by fluorescence microscopy analysis. (**B**) Percentage of EdU-positive cells. (**C**) Flow cytometric analysis of the cell cycle. (**D**) Proportions of ovarian GCs in the G1, S, and G2 phases. (**E**) Proliferation index calculated based on the percentages of cells in S and G2/M phases. (**F**) mRNA levels of proliferation-related markers. (**G**,**H**) Expression levels of proliferation-associated proteins. Data are mean ± SD. *n* = 5 independent experiments. One-way ANOVA followed by Tukey’s test. Different lowercase letters represent significant differences between groups (*p* < 0.05). ** *p* < 0.01; *** *p* < 0.001.

We further investigated how GPS mitigates ERS-induced loss of ovarian GCs. EdU assay showed that Tm markedly inhibited GC proliferation, while GPS treatment effectively alleviated this inhibition (Figure 3A,B). Flow cytometric cell cycle analysis revealed that Tm induced S-phase arrest, which was relieved by GPS, promoting GC proliferation (*p* < 0.05; Figure 3C–E). RT-qPCR analysis showed that Tm significantly reduced the transcript levels of *CDK2*, *PCNA*, *PI3K*, and *AKT*, and GPS attenuated these decreases (*p* < 0.05; Figure 3F). Western blot results confirmed that Tm reduced CDK2, PCNA, p-PI3K, and p-AKT/AKT protein levels, and GPS pretreatment reversed these effects (*p* < 0.05; Figure 3G,H). These findings suggest that GPS mitigates ERS-induced GC damage and activates the PI3K/AKT signalling pathway.

**Figure 4:**
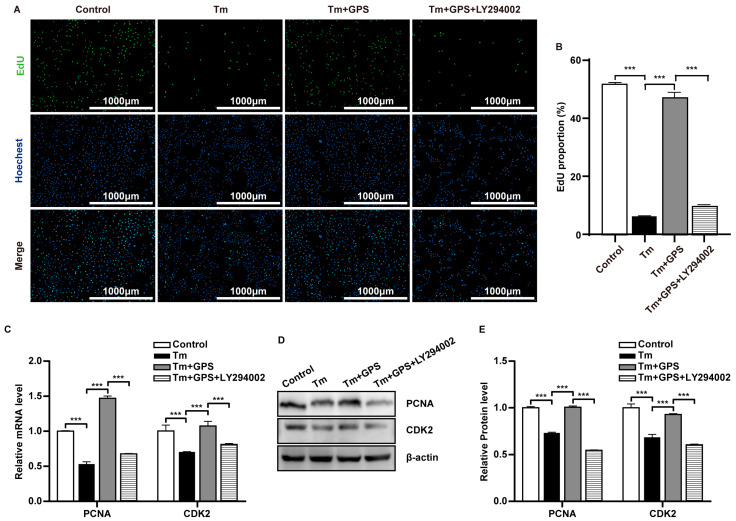
Ginseng polysaccharides alleviate ERS-induced ovarian GC proliferation impairment via the PI3K/AKT signalling pathway. (**A**) EdU staining of cells followed by fluorescence microscopy analysis. (**B**) Proportion of cells exhibiting EdU positivity. (**C**) The mRNA levels of proliferation-related markers. (**D**,**E**) Expression levels of proliferation-associated proteins. Data are mean ± SD. *n* = 5 independent experiments. One-way ANOVA followed by Tukey’s test. *** *p* < 0.001.

To further verify whether GPS acts through the PI3K/AKT pathway, we used the PI3K inhibitor LY294002. EdU assays showed that LY294002 suppressed the pro-proliferative effect of GPS (*p* < 0.05; Figure 4A,B). Moreover, LY294002 downregulated the mRNA and protein expression of *PCNA* and *CDK2* (*p* < 0.05; Figure 4C–E). These data demonstrate that GPS promotes the recovery of ovarian GC proliferation following ERS-induced injury in a PI3K/AKT-dependent manner.

### 3.4. Ginseng Polysaccharides Prevent Tm-Induced Impairment of Estrogen Secretion via a PI3K/AKT-Dependent Mechanism

**Figure 5:**
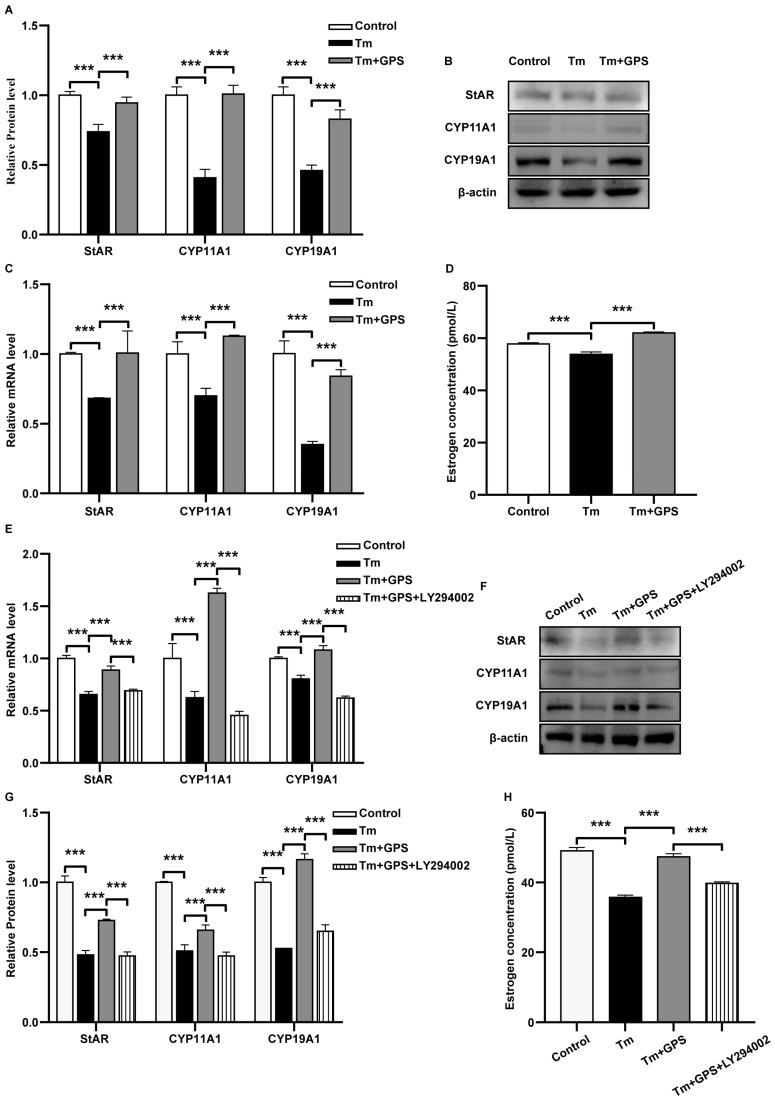
Ginseng polysaccharides prevent Tm-induced estrogen secretion impairment via a PI3K/Akt-dependent mechanism. (**A**) mRNA levels of estrogen-synthesis-related markers. (**B**,**C**) Expression levels of estrogen-synthesis-related proteins. (**D**) Estradiol (E2) content in ovarian GCs. (**E**) mRNA levels of estrogen-synthesis-related markers under inhibitor treatment. (**F**,**G**) Expression levels of estrogen-synthesis-related proteins under inhibitor treatment. (**H**) Estradiol (E2) content in ovarian GCs. Data are mean ± SD. *n* = 5 independent experiments. One-way ANOVA followed by Tukey’s test. *** *p* < 0.001.

We next examined the effects of GPS on Tm-induced disruptions in estrogen production. RT-qPCR and Western blot analyses showed that Tm significantly reduced the mRNA and protein expression of *StAR*, *CYP11A1*, and *CYP19A1* (*p* < 0.05; Figure 5A–C). GPS pretreatment alleviated these inhibitory effects. ELISA results indicated that Tm suppressed estradiol secretion, and GPS restored E2 levels to those comparable to the control (Figure 5D). The beneficial effects of GPS on estrogen synthesis and secretion were abolished by LY294002 (*p* < 0.05; Figure 5E–H). These findings demonstrate that GPS alleviates Tm-induced estrogen secretion dysfunction through PI3K/AKT pathway activation.

### 3.5. GPS Alleviates ER Stress-Induced Ovarian Injury In Vivo

**Figure 6:**
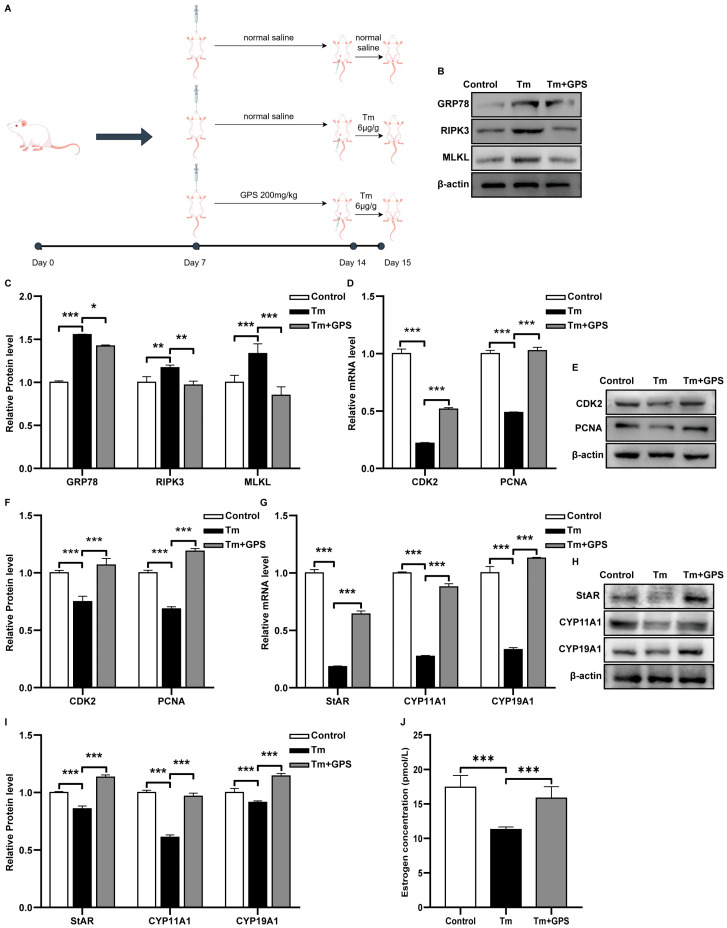
GPS alleviates ERS-induced ovarian injury in vivo. (**A**) Schematic diagram of the animal experiment. (**B**,**C**) Expression levels of ER stress- and necroptosis-related proteins. (**D**) mRNA levels of proliferation-related markers. (**E**,**F**) Expression levels of proliferation-associated proteins. (**G**) mRNA levels of estrogen-synthesis-related markers. (**H**,**I**) Expression levels of estrogen-synthesis-associated proteins. (**J**) Serum estradiol (E2) levels. Data are mean ± SD. *n* = 6 mice per group. One-way ANOVA followed by Tukey’s test. * *p* < 0.05; ** *p* < 0.01; *** *p* < 0.001.

To evaluate the protective effects of GPS in vivo, we established an ERS model in mice via intraperitoneal injection of Tm. GPS was administered orally, and its efficacy was assessed by comparing the GPS-pretreated group with the Tm-only group (Figure 6A). RT-qPCR and Western blot analyses revealed that Tm upregulated key markers of ERS and necroptosis in mouse ovaries, and GPS significantly mitigated these effects (*p* < 0.05; Figure 6B,C). ERS suppressed the level of expression of the proliferation-related markers *PCNA* and *CDK2*, and GPS pretreatment reversed these changes (*p* < 0.05; Figure 6D–F). Furthermore, GPS administration enhanced the levels of expression for steroidogenic enzymes *StAR*, *CYP11A1*, and *CYP19A1* (*p* < 0.05; Figure 6G–I) and restored serum E2 levels (Figure 6J). These in vivo results demonstrate that GPS effectively alleviates ERS-induced ovarian injury.

## 4. Discussion

To elucidate the protective mechanism of ginseng polysaccharides (GPSs), which mitigates necroptotic cell death triggered by ERS in bovine ovarian GCs, this study systematically investigated the process using bovine ovarian GCs as an in vitro model. Receptor-interacting protein kinase 3 (RIPK3), a central regulator of necroptosis, initiates the conserved RIPK3-MLKL signalling cascade in mammalian cells by promoting the phosphorylation and membrane translocation of mixed-lineage kinase domain-like protein (MLKL) [29,30,31]. In this study, we employed tunicamycin (Tm), a classical ERS inducer, to establish the cellular model. Tm triggers ERS by promoting the buildup of misfolded proteins and triggering the UPR [32]. Consistently with previous findings [17], treatment with 5 μg/mL Tm significantly reduced the viability of ovarian GCs and induced necroptosis. This concentration effectively activated the expression of RIPK3 and MLKL proteins in bovine ovarian GCs, promoting necroptosis, whereas GPS pretreatment markedly reversed this process. Notably, necroptosis is often accompanied by the release of pro-inflammatory cytokines [33]. GPS has been demonstrated to suppress the secretion of these pro-inflammatory factors [34,35] and exhibit potent antioxidant activity to alleviate oxidative stress damage [23,36]; whether GPS directly regulates the RIPK3-MLKL pathway requires further experimental validation. Furthermore, given the close association between ERS-induced necroptosis and aberrant UPR activation, the potential synergistic protective effects of GPS via modulation of UPR signalling warrant deeper investigation.

The UPR serves as a key adaptive mechanism enabling cells to cope with ERS, determining cell fate through regulating of genes involved in the secretory pathway. Its activation depends on three core transmembrane sensors: IRE1, PERK, and ATF6 [32]. Under homeostasis, the ER chaperone BiP/GRP78 binds to these sensors, maintaining them in an inactive state. During ERS, GRP78 dissociates to bind unfolded proteins, thereby releasing the sensors and initiating downstream signalling [37]. When ERS exceeds the cellular repair capacity, persistent abnormalities—such as upregulation of GRP78, phosphorylation of IRE1/PERK, and cleavage activation of ATF6—ultimately lead to cell death [10]. Han et al. [24] reported that GPS suppressed Toxoplasma gondii-induced ERS in testicular cells and alleviated ERS-related testicular toxicity. Similarly, our results showed that GPS downregulated GRP78 expression and inhibited the activation of PERK, IRE1, and ATF6 in Tm-treated GCs, suggesting that GPS may alleviate excessive ERS activation rather than directly suppressing the ERS process. Under ERS conditions, aberrant activation of the PERK-ATF4 pathway serves as a critical link connecting UPR to necroptosis [17]. Previous studies have indicated that GPS can block this pathway by inhibiting PERK protein expression [22]. This mechanism was further validated in our study, suggesting that targeting the PERK-ATF4 pathway to disrupt the transmission of ERS signals to necroptosis is one of the core mechanisms by which GPS reduces necroptosis in bovine ovarian GCs. It should be noted that the three branches of UPR (IRE1/XBP1, PERK/ATF4, ATF6) exhibit complex synergistic and antagonistic relationships during ERS [38,39,40]. The molecular mechanisms by which GPS precisely regulates the balance among these branches to restore ER homeostasis remain unclear and will be a focus of our future research.

Given that ERS also potently suppresses cell proliferation, we next investigated whether GPS could counteract this effect and restore proliferation in bovine ovarian GCs. Cyclin-dependent kinase 2 (CDK2), as a core cell cycle kinase, phosphorylates downstream substrates to drive cell cycle progression and plays a pivotal role, specifically during the activation of the S phase for DNA synthesis [41,42,43]. It has been reported that ERS can disrupt cell cycle homeostasis by modulating the expression of cyclin D, leading to reduced cell viability [44]. Similarly, our results showed that Tm-induced ERS significantly inhibited the proliferation of bovine ovarian GCs, causing cell cycle arrest in the S phase. GPS pretreatment markedly upregulated CDK2 protein expression, promoted S phase progression, increased the proportion of EdU-positive cells, and effectively restored the proliferative capacity of ovarian GCs. Notably, the PI3K/Akt signalling pathway is a key regulator of bovine ovarian GC proliferation and function [45,46,47], and previous studies have confirmed that GPS can modulate necroptosis through the RIP1/RIP3/MLKL signalling axis [48,49,50]. In our study, the proliferative protective effect of GPS was completely abolished by a PI3K/Akt inhibitor in our study. This finding indicates that GPS-mediated restoration of cell cycle progression is dependent on PI3K/Akt pathway activation, which may upregulate CDK2 expression. In parallel, GPS treatment was associated with reduced activation of the RIPK3-MLKL necroptotic pathway; however, whether this inhibitory effect is directly mediated by PI3K/Akt remains to be determined. Collectively, these observations imply that GPS may exert a dual protective role via the PI3K/Akt pathway—restoring cell proliferation and potentially mitigating necroptosis.

Beyond its impact on proliferation, ERS is also known to impair the endocrine function of ovarian GCs, including the secretion of estrogen [51]—a key regulatory factor in follicular development and atresia, as healthy follicle maturation depends on adequate estrogen levels [52,53]. Estrogen synthesis is precisely regulated by key steroidogenic enzymes (e.g., CYP19A1) and hormone receptors [29,54]. ERS has been identified as a significant pathological factor that suppresses steroid hormone synthesis in ovarian GCs [55,56]. Studies have shown that ERS can reduce serum estradiol concentrations in rats [57] and inhibit progesterone production and the expression of steroidogenic enzymes in goat GC lines [58]. Consistently, we observed that Tm-induced ERS significantly suppressed estrogen secretion in bovine ovarian GCs. Notably, GPS effectively upregulated the expression of steroidogenesis-related markers, such as *StAR*, *CYP11A1*, and *CYP19A1*, alleviating impaired estrogen secretion. Importantly, this protective effect of GPS was completely abolished by a PI3K inhibitor, indicating that GPS-mediated improvement of ERS-impaired steroidogenesis is also dependent on PI3K/Akt pathway activation—a finding that aligns with previous reports [24]. Collectively, these results suggest that the PI3K/Akt pathway may serve as a central hub for GPS to exert its protective effects on GCs, by regulating both cell proliferation and steroid hormone synthesis.

Multiple in vivo studies have supported that dietary supplementation with GPS has no adverse effects on maternal reproductive performance and can enhance maternal immune and antioxidant capacities [59,60]. In a D-galactose-induced mouse model of premature ovarian insufficiency, Shang et al. [61] found that GPS specifically increased estradiol (E2) levels, decreased follicle-stimulating hormone (FSH) levels, and ameliorated pathological damage associated with ovarian insufficiency by inhibiting oxidative stress and granulosa cell apoptosis. Consistent with this, our study further confirmed in a tunicamycin (Tm)-induced mouse model of ovarian ERS that GPS alleviated ERS-mediated disturbances in estrogen secretion, restored the mRNA and protein levels of crucial steroidogenic enzymes, reversed the decline in cell proliferation-related indicators, and inhibited GC necroptosis. These in vivo results corroborate the findings from our in vitro experiments on bovine ovarian GCs. Notably, GPS downregulated GRP78 expression in mouse ovarian tissue. This result is highly consistent with the observed downregulation of GRP78 expression in vitro, providing cross-validation between in vivo and in vitro experiments. These findings suggest that GPS also has the potential to regulate ovarian reproductive cell function in vivo and support the research conclusion proposed by Han et al. [24] that “GPS exerts organ-protective effects by modulating the ERS pathway in vivo,” further reinforcing the core protective mechanism of GPS: “inhibiting excessive ERS activation–restoring cell proliferation–improving hormone synthesis.” Collectively, these cross-species results support the potential of GPS to regulate ovarian function in vivo by alleviating excessive ERS activation. While these findings provide preclinical evidence for the potential application of GPS in ERS-related ovarian dysfunction, further studies are required to evaluate its efficacy and safety before extrapolating to clinical settings.

This study confirmed through in vitro experiments that GPS protects against Tm-induced ovarian dysfunction by inhibiting the PI3K/Akt pathway, while in vivo experiments demonstrated its efficacy in alleviating ERS-related ovarian damage. However, how the PI3K/Akt pathway precisely distinguishes specific molecular targets involved in proliferation regulation, necroptosis inhibition, and steroidogenesis improvement, as well as the synergistic balance mechanism of GPS among the three UPR branches, remains unclear and will be the focus of subsequent research.

## 5. Conclusions

**Figure 7:**
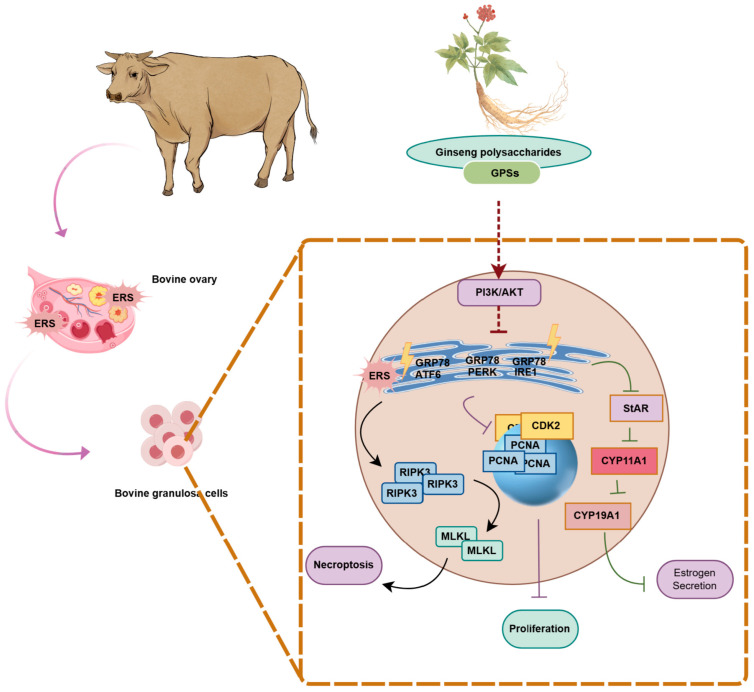
Schematic diagram of the mechanism of action of GPS in ERS-induced damage of bovine ovarian granulosa cells.

In summary, we reported that GPSs alleviate ERS-induced damage in bovine GCs and ameliorate ovarian dysfunction in ERS-challenged mice. Mechanistically, GPS-mediated protection is dependent on activating the PI3K/Akt pathway, which restores cell cycle progression, enhances steroid hormone synthesis, and potentially inhibits ERS-driven necroptosis (Figure 7).

In vivo experiments cross-validated these in vitro findings. However, the specific downstream targets of PI3K/Akt and the regulatory mechanism of GPS on UPR branches remain to be explored. This work provides preclinical evidence for GPS application in ERS-related ovarian disorders.

## Figures and Tables

**Table 1:** Sequence of primers used for RT-qPCR.

Genes	Species	Primer Sequences (5′–3′)	Annealing Temperature, °C	Accession Number
*PCNA*	Bovine	F: AAGCCACTCCACTGTCTCCTAC	61	NM_001034494.1
		R: TCCTTCTTCATCCTCGATCTTGGG		
*CDK2*	Bovine	F: CGCTCACTGGCATTCCTCTTC	61	NM_001014934.1
		R: GGACCCATCTGCGTTGATAAGC		
*PI3K*	Bovine	F: TGGTGACAAGTGGTGGGACAG	58	NM_001206047.2
		R: AGGTGTAAGTGCCATCTGGTAGC		
*AKT*	Bovine	F: TAAGCAGAAGCCTATCACTCCAGAG	60	NM_173986.2
		R: GCAGAGCACTCCACATACTTGAC		
*CYP11A1*	Bovine	F: GTCAAAGCCTGCCCACCCATC	61	NM_176644.2
		R: ATGCCAGCTCCCTCTCCAGTG		
*CYP19A1*	Bovine	F: GTCGTCCTGGTCACCCTTCTG	61	NM_174305.1
		R: GGTCTCTGGTCTCGTCTGGATG		
*STAR*	Bovine	F: ATGGTGCTCCGCCCCTTGG	61	NM_174189.3
		R: TCTGCGAGAGGACCTGGTTGATG		
*RIPK3*	Bovine	F:GTCCACATTTCAGGGAGGCT	56	NM_001435166.1
		R:GAAGGATCCCAGAGTCTGTCT		
*MLKL*	Bovine	F:GGGAGCAGCACTTCTCTGTTA	62	XM_059877358.1
		R:GGGGCTGCTAAGTCACAATG		
*GRP78*	Bovine	F:CGTGCGTTTGAGAGCTCAGT	58	NM_001075148.1
		R:TAGGGCTTCGCAGGAAAACC		
*PERK*	Bovine	F:CCAGCAAAGAGGAGCCCAGAATG	60	NM_174584.3
		R:AAGTGGTTGGTCTTGACGGAGAAAC		
*IRE1*	Bovine	F:CCGAAGTTCAGATGGCATTC	56	NM_001077828.1
		R:TCTGCAAAGGCTGATGACAG		
*ATF6*	Bovine	F:GGATTTGATGCCTTGGGAGACAG	59	XM_024989876.2
		R:TGAGGAGATGAGATTGAACAACTTGAG		
*β-actin*	Bovine	F: TTGATCTTCATTGTGCTGGGTG	60	NM_174226.2
		R: CTTCCTGGGCATGGAATCCT		
*PCNA*	Mouse	F:GAAGTTTTCTGCAAGTGGAGAG	54	NM_011045.2
		R:CAGGCTCATTCATCTCTATGGT		
*CDK2*	Mouse	F:CCAGGAGTTACTTCTATGCCTGA	56	NM_001428414.1
		R:TTCATCCAGGGGAGGTACAAC		
*STAR*	Mouse	F:CGGGTCTTGGGTCCTGGATTGT	62	NM_011485.5
		R:GCTGCCCTCGCTCACCTTAAG		
*CYP11A1*	Mouse	F:GCTCAACCTGCCTCCAGACTTC	61	NM_011045.2
		R:CCTCCTGCCAGCATCTCGGTAA		
*CYP19A1*	Mouse	F:TCGTCGCAGAGTATCCAGAGGT	60	NM_001348171.1
		R:CGCATGACCAAGTCCACAACAG		
*GRP78*	Mouse	F:CTGGCCGAGACAACACTGACCT	63	NM_001163434.1
		R:GCGACGACGGTTCTGGTCTCAC		
*β-actin*	Mouse	F:GTCTTCCCCTCCATCGTG	60	NM_007393.5
		R:AGGGTGAGGATGCCTCTCTT		

## Data Availability

The data presented in this study are available on request from the corresponding author.
